# Hallmarks of Comparative Transcriptome between Rhizomorphs and Hyphae of *Armillaria* sp. 541 Participating in Fungal Symbiosis with Emphasis on LysM Domains

**DOI:** 10.3390/microorganisms11081914

**Published:** 2023-07-27

**Authors:** Bing Li, Liu Liu, Dawei Zhang, Shunxing Guo

**Affiliations:** 1 Institute of Medicinal Plant Development, Chinese Academy of Medical Sciences & Peking Union Medical College, Beijing 100193, China; zudengtianxia@126.com (B.L.); liuliu0026@foxmail.com (L.L.); 2 Institute of Bioinformatics and Medical Engineering, School of Electrical and Information Engineering, Jiangsu University of Technology, Changzhou 213001, China

**Keywords:** *Armillaria* sp., rhizomorph, LysM domain, *aLDRG*, chitinoligosaccharide

## Abstract

*Armillaria* sp. 541, a genus of root-infecting fungi, forms a symbiosis with traditional Chinese medicine *Gastrodia elata* (Orchid) and *Polyporus umbellatus* via extensive networks of durable rhizomorphs. It is not clear the hallmarks of comparative transcriptome between the rhizomorphs and hyphae of *Armillaria* sp. 541. In the present study, transcriptomic analysis of *Armillaria* sp. 541 identified 475 differentially expressed genes (DEGs) between *Armillaria* rhizomorphs (AR) and hyphae (AH). Of them, 285 genes were upregulated and 190 were downregulated. Bioinformatics analyses and tests demonstrated DEGs involved in oxidoreductase activity and peptidoglycan binding were significantly enriched in this process when rhizomorph formed from hyphae. We accordingly obtained 14 gene-encoding proteins containing the LysM domain, and further consensus pattern and phylogenetic analysis indicated that their amino acid sequences were conserved and their biological functions may be peptidoglycan binding for recognition between the fungus and host. Among these genes, one, named *Armillaria LysM domain recognition gene* (*aLDRG*), was expressed significantly when rhizomorphs were differentiated from hyphae. It was located in the cortical cells of the rhizomorph by in situ hybridization. Furthermore, biolayer interferometry (BLI) assay demonstrated that aLDRG can bind specifically to chitin oligosaccharide of the fungal cell wall, including *N*,*N*′,*N*″-Triacetylchitotriose (CO3) and *N*,*N*′,*N*″,*N*′″,*N*″″-Pentaacetylchitopentaose (CO5). Therefore, we deduced that *Armillaria* sp. 541 expressed higher levels of LysM protein aLDRG for better binding of oligosaccharide after rhizomorphs were generated. This study provides functional genes for further studies on the interaction between *Armillaria* sp. 541 and its host.

## 1. Introduction

*Armillaria* spp. belongs to the Physalacriaceae family of the order Agaricales and is widely distributed throughout the world, from Europe to East Asia and to North America [1]. They are types of facultative parasitic fungi characterized by edible sporocarps and medicinal function. The genus *Armillaria* is mainly considered to comprise facultative necrotrophs, characterized by both parasitic and saprotrophic phases. For saprotrophic species, known as forest destruents, *Armillaria* can effectively degrade plant cell-wall components (such as lignin, cellulose, etc.), and their degradation products could be used as soil humus to provide nutrients for other species in the forest [2]. As parasitic fungi, *Armillaria* can parasitize more than 500 tree species causing root rot, leading to potentially substantial economic losses [2]. Interestingly, *Armillaria mellea*, one of these fungi, has also been observed to establish a symbiotic relationship with the original plant of traditional Chinese medicine (TCM), such as *Gastrodia elata* (an orchid) and the fungus *Polyporus umbellatus* (sclerotia) [3,4]. Without *Armillaria*, *G. elata* could not grow at all [5], which would lead to over ten billion economic losses, as was the case with *P. umbellatus.* Under natural conditions, the growth and development of *P. umbellatus* is dependent on *Armillaria*. Under suitable conditions, *Armillaria* produces fruiting bodies with yellow-capped and fungus-screen rings. The fruiting body of some *Armillaria* is delicious and nutritious, and it is widely eaten across the world. Active components, such as amino acids, polysaccharides, adenosines and sesquiterpenoids, have been isolated and identified from *Armillaria.* These components have various biological activities, such as anti-aging, hypnosis, anti-convulsion, improvement in heart and brain blood circulation, immune regulation, etc. [6]. Drugs containing *Armillaria* mycelia or its extract, such as the Yun Tong Ding capsule, Nao Xin Shu oral liquid, etc., are widely used in clinics. What is even more surprising is that *Armillaria* sp. holds the title of the world’s largest known organism [7,8]. A strain of *Armillaria ostoyae* was found in Michigan, and it covered 37 hectares and weighed about 400 tons. According to Ferguson et al. 2003, in the Blue Mountains (Oregon, USA), *A.ostoyae* covers 900 hectares as an organism and is more than 2000 years old [9]. Almost all of these properties or applications are based on one type of structure called rhizomorph, which can create a bridge between food sources and further expand the range of *Armillaria*.

Studies highlighted the important functional roles of *Armillaria* rhizomorphs in withstanding adverse environments, searching for nutrients and penetrating hosts. Rhizomorphs can persist for decades and respond opportunistically to spatially and temporally changing environments by forming extensive and durable rhizomorph networks [7,10]. In addition, rhizomorphs spread throughout the soil in search of nutrients derived from both decaying wood and living tree roots [11,12]. Importantly, the rhizomorphs of *Armillaria* have similarly been characterized as functional structures during the colonization of *G. elata* (Orchid) protocorm and *P. umbellatus* sclerotia [3,4]. Our previous studies indicated that metabolic pathways involved in steroids, etc., were active and enriched during the infection of *P. umbellatus* sclerotia by *A. mellea* by metabolic and transcriptomic analysis. In comparison with the control group, the content of polyporusterone A, polyporusterone B and ergosterol in the infected sclerotia increased by 75.0%, 20.0% and 32.2%, respectively [4]. If the rhizomorph network of *Armillaria* linked with *P. umbellatus* is cut, the sclerotia will stop growing and developing [13]. Therefore, it is obvious that rhizomorphs are the tissue where *Armillaria* sp. performs those biological functions. 

Some studies have sought to identify the genes or proteins that carry out the biological functions of rhizomorphs. Collins et al. [14] reported 739 mycelia (mixed rhizomorphs) proteins in *A. mellea*, and the analysis revealed a rich reservoir of carbohydrate esterase (CE) families, carbohydrate-binding modules (CBMs), glycoside hydrolases (GH), laccases and peroxidases, etc., for carbon substrate degradation. The presence or expression level of these enzymes was also affected by external conditions. The transcriptome of *A. sinapina* was performed between that supplemented or not supplemented with betulin mycelia, and the bioinformatics analysis showed that gene transcripts associated with the degradation of cell walls were significantly upregulated in the betulin treatment group, including glucosidases, invertases, laccases, glucanases, mannosidases, debranching enzymes and peroxidases [15]. In addition, the protein expression profiles between rhizomorphs and hyphae were also different. Sahu et al. [16] identified 602 and 612 transporters in *A. cepistipes* and *A. ostoyae*, respectively, based on conserved domains. In rhizomorphs and hyphae, there were 120 and 100 upregulated transporters, respectively, and much fewer downregulated transporters in these two species. Then, they speculated that rhizomorphs might be involved in the transfer of the intermediates for decomposition (such as sugar) between *Armillaria* and host or different parts of the colony. A recent study showed that symbiotic and saprotrophic *Armillaria* had significant differences in genomic information. Cai et al. [17] conducted whole-genome sequencing of the *A. gallica* Jzi34 strain symbiotic with *Gastrodia elata*, and the analysis results showed that it was significantly contracted for the carbohydrate enzyme gene family, while the number of glycosyl transferase (GT) genes accounted for the largest proportion. In addition, it also had an expansion of genes encoding cytochrome P450 and auxiliary activity enzymes AA3-2 [17]. *Armillaria* sp. 541 is a symbiotic fungus with *Polyporus umbellatus*, and it can result in good yield for *P. umbellatus* with increased active ingredient content [4]. However, little is known about the functional genes involved in rhizomorph’s generation.

Among the many functional genes reported in *Armillaria*, genes encoding LysM play an important role in the fungus interaction with the host. Sipos et al. [18] found that certain chitin-binding genes were duplicated in rhizomorphs compared with mycelium. In these genes, types of Pfams, including LysM, were over-represented. LysMs are a family of carbohydrate-binding modules with a βααβ structure and a length of about 50 amino acids. It has been confirmed that they are universally found in all life except for Archaea [19]. LysM is associated with chitin during the interaction between the host and fungi. Chitin is a characteristic component of fungi that distinguishes them from host plants, and chitin oligomers (Glc-NAc oligomers) digested from chitin are microbe-associated molecular patterns (MAMPs) that activate pattern-triggered immunity (PTI) in plants [20]. During the interaction between the plant and fungus, plants secrete chitinases to hydrolyze chitin in the fungal cell wall, resulting in chitin oligomers. These oligomers can be subsequently sensed by plant chitin receptor complexes (containing LysM) to initiate intracellular immune signaling [21,22]. To evade the immune response, fungi have evolved types of mechanisms that can infect plants. One of the approaches is to secrete LysM effectors that competitively bind to Glc-NAc oligomers on the surface of fungi and disguise themselves to evade host chitinases degradation, leading to the blockage of PTI and an increase in pathogenicity [23,24]. Therefore, it is not difficult to conclude that LysM and LysM binding to Glc-NAc oligomers are essential during fungus interactions with its host. However, no relevant studies have been conducted on *Armillaria*. 

Therefore, we are interested in studying the specialized rhizomorphs that are both morphologically and functionally complex. In this study, the transcriptome was sequenced for hyphae differentiation to form rhizomorphs of *Armillaria* sp. 541, which was symbiotic with *P. umbellatus*, and a series of genes differentially expressed in this process were obtained. Fourteen of these genes encoding LysM-containing proteins were shown to likely be involved in host interactions. Further functional validation experiments showed affinity profiles of *aLDRG* and products of chitin, and it was hypothesized that *aLDRG* in *Armillaria* sp. 541 was associated with host symbiosis. This study provides functional genes for further studies of symbiosis between *Armillaria* sp. 541 and its host, and it can also be useful to determine the pathogenicity of other species and isolates.

## 2. Materials and Methods

### 2.1. Strain and Culture of Armillaria on Plates

For the purposes of the RNA sequence test and verification, we used one strain named *Armillaria* sp. 541, which was isolated from the sclerotia of *P. umbellatus* collected in the field and subsequently rejuvenated and cultured as previously described [25]. It was preserved in the China General Microbiological Culture Collection Center (No. 21080). *Armillaria* sp. 541 was inoculated on a Petri dish containing potato dextrose agar (PDA) and cultured in the dark at 25 °C. On day 28, the hyphae and rhizomorphs of this fungus growing on the same plate were defined as *Armillaria* hyphae (AH, as the control group) and rhizomorphs (AR, as the test group), respectively. Material of AH and AR were collected and rapidly frozen in liquid nitrogen for subsequent transcriptomic and quantitative real-time PCR (qRT-PCR) tests. Three biological replicates were obtained for each group. Fresh rhizomorphs were obtained for in situ hybridization.

### 2.2. RNA-Seq for Armillaria sp. 541

RNA-Seq was performed by Allwegene Technology Co., Ltd. (Beijing, China). Total RNA was isolated from the samples of AH and AR using an RNeasy plant mini kit (QIAGEN, Inc., Valencia, CA, USA) and treated with DNase I (Roche, Inc., Branchburg, NJ, USA) to eliminate contaminating DNA, according to the instructions. The integrity of RNA was verified by using an Agilent 2100 Bioanalyzer (Agilent Technologies, Inc., Santa Clara, CA, USA). The mRNA content of samples was selectively enriched using magnetic Oligo (dT) beads, and a random hexamer was then used to target the mRNA as a template to synthesize the first-strand cDNA. Having obtained the first-strand cDNA, we proceeded to synthesize second-strand cDNA following the addition of DNA polymerase I, buffer, and dNTPs. AMPure XP beads were used to purify the resulting double-stranded cDNA, following the end repair and the addition of A-tails, and adapter sequences. Subsequently, double-stranded cDNA was size-selected via AMPure XP beads, and the cDNA library was constructed by PCR amplification. The sequencing of the cDNA to obtain raw read pairs was performed using the Illumina second-generation high-throughput sequencing platform based on the PE150 sequencing strategy.

### 2.3. Transcriptomic Analysis 

Low-quality reads, namely adapter sequences with >10% unknown nucleotides and those with Q20 values < 20% (percentage of sequences with sequencing error rates < 1%), were removed using Trimmomatic v0.33 [25]. Clean reads were mapped to the *Armillaria gallica* (taxid47427) genome [18] using Spliced Transcripts Alignments to Reference (STAR) software after filtering the raw reads [26]. The expression levels of genes were estimated on the basis of FPKM (fragments per kilobase of exon per million mapped fragments) values determined using Cufflinks software [27].

### 2.4. Differentially Expressed Genes and Unannotated Transcripts 

Principal component analysis (PCA) is a technology that simplifies data analysis by reflecting the differences in multiple sets on two-dimensional coordinate diagrams [28]. To preliminarily judge whether there were differences between AR and AH, PCA analysis was performed.

DESeq [29] was used to evaluate the differential expression of genes between the AH and AR structures of *Armillaria* sp. 541. On the basis of the relative ratios of the FPKM values (FPKM > 1), the differences in gene abundance between the two tissue types were calculated. Only genes with a q-value < 0.05 and an absolute value of a log2 fold change ≥ 1 were considered as differentially expressed and used for subsequent analyses. The biological functions of differentially expressed genes (DEGs) were determined using Gene Ontology (GO) and the Kyoto Encyclopedia of Genes and Genomes (KEGG).

The coding regions of unannotated intergenic transcripts, the class code of which was designated as “Novel” in the Cuffcompare output, were predicted using Cufflinks version 2.1.1 [27]. 

### 2.5. Transcriptional Profiles of Genes Encoding LysM Proteins in Armillaria sp. 541

To determine the genes that encode LysM domain-containing proteins in *Armillaria* sp. 541, we initially constructed a LysM domain database of fungi using the following procedure. (1) Reference LysM domains were downloaded from an online network (http://www.cazy.org/CBM50.html, accessed on 28 April 2023), which were classified as a carbohydrate-binding module family (CBM) of 50 proteins. (2) Proteins containing a LysM domain were selected specifically from fungi. (3) Only those proteins that had been annotated and deposited in the Swiss-Prot database were selected. The transcriptional profiles of genes encoding LysM proteins in *Armillaria* sp. 541 were obtained using the NCBI local BLASTP tool (https://blast.ncbi.nlm.nih.gov/Blast.cgi, accessed on 28 April 2023) to screen the LysM domain database of fungi. The sequences of these genes were further analyzed using the Pfam online tool (http://pfam.xfam.org/, accessed on 28 April 2023) to validate and select the final LysM domains [30] in *Armillaria* sp. 541, and basic gene parameters were predicted by using the online tool ProtParam (https://web.expasy.org/protparam/, accessed on 28 April 2023), SingnalP 5.0 server (http://www.cbs.dtu.dk/services/SignalP/, accessed on 28 April 2023) and SMART (http://smart.embl-heidelberg.de/, accessed on 28 April 2023) [31,32]. Gapped local alignments of *Armillaria* sp. 541 LysM motifs were generated using GLAM2 (http://web.mit.edu/meme_v4.11.4/share/doc/glam2.html, accessed on 28 April 2023) [33]. LysM domain-containing genes obtained from the fungal database and those identified in *Armillaria* sp. 541 were combined for phylogenetic analysis using MEGA11.0 based on the maximum likelihood model and 500 bootstrap replications [34]. SWISS-MODEL provided structures of all these 14 proteins [35]. Proteins that contained LysM Motif and met the following criteria were defined as LysM effectors. They possessed signal peptides and only contained LysM domains with a characteristic βααβ spatial structure [36].

### 2.6. qRT-PCR Validation of the Transcriptomic Data 

To verify the results of transcriptome analysis, we selected 6 DEGs for validation by qRT-PCR. The samples of mycelia and rhizomorphs conducted for qRT-PCR were the same as those for RNA-Seq, and the primers used are presented in Supplementary Table S1. qRT-PCR was performed following our previously published methods [37] and the major procedures as follows: Reverse transcription synthesis of cDNA was performed using 500 ng total RNA for each sample according to the PrimeScript^TM^ RT Reagent Kit (Takara, Kusatsu, Japan). cDNA was then diluted 10-fold for qRT-PCR reaction. Reactions were performed by the Light Cycler^®^ 480II (Roche, Basel, Switzerland) software and the real-time fluorescent quantitative PCR instrument, and each PCR mixture (15 µL) contained 0.3 µL of each forward and reverse gene-specific primer: 7.5 μL 2× SYBR^®^ Premix Ex Taq^TM^ (Takara, Kusatsu, Japan), 1 µL of cDNA and 5.9 µL of sterile ddH_2_O. The PCR cycle consisted of an initial denaturation step of 95 °C for 30 s, followed by 40 cycles of 95 °C for 5 s and 60 °C for 20 s. EF-1γ was used as the endogenous control. Three samples from each group were analyzed, and each sample was repeated in triplicate. The relative gene expression was calculated by following the 2^−ΔΔCt^ method.

### 2.7. In Situ Hybridization 

A primer synthesis corresponding to the targeted gene, with the sequence 5′-DIG-CGGTGTAATTGCGAGCACAGTTAGCCACTTGGG-DIG-3′ (theoretical molecular weight 9083.23) was carried out by ZCIBIO Technology Co., LTD. The solid-phase phosphoramidite method was introduced into synthesizing DIG-probe, which consisted of detritylation, coupling, capping and oxidation procedures per cycle, performed by OligoMaker 48 (Frederiksberg, Denmark). After that, the oligonucleotide was chemically cleaved from the solid carrier (CPG) using 25% ammonia, and the protective group was removed by trichloroacetic acid (TCA). The crude product was purified using high-performance liquid chromatography, and the molecular weight of the purified fraction was measured by an LTQ-MS mass spectrometer. In situ hybridization of the *Armillaria LysM domain-recognition gene* (*aLDRG*) was performed as previously described [38]. White tips and branches of rhizomorphs were harvested and fixed with FAA fixative solution (Solarbio Life Sciences, Beijing, China) at 4 °C for 12 h. Then, the fixed materials were dehydrated with gradient ethanol solution (4 °C) in a range of ethanol from 30–100% for 30 min and then imbedded in paraffin. After that, imbedded tissue was cut into pieces and digested with proteinase K (final concentration 20 μg/mL) at 37 °C for 18 min. Pieces were incubated in 1.0 uM *aLDRG* probe solution overnight at 42 °C, and then blocked with a rabbit serum at 25 °C for 1 h. Subsequently, pieces were incubated in the anti-DIG-HRP solution (Solarbio Life Science, Beijing, China) (1:5000) at 37 °C for 50 min. Finally, diamino benzidine (DAB) was added, and the binding of the probe was examined under a microscope.

### 2.8. Expression and Purification of Recombinant aLDRG 

The *aLDRG* gene was chemically synthesized and expressed in *Escherichia coli* after codon optimization by Genewiz, Inc. (Suzhou, China) excluded the sequence encoding of the first 20 residues, which was predicted to be the signal peptide as determined by the SIGNALP 4.1 Server [39]. The resulting sequence was subcloned into NdeI-BamHI sites of a pET28a vector, and the recombinant protein was expressed in *E. coli* BL21 (DE3). Overnight culture in LB medium supplemented with kanamycin (100 μg/mL) was sub-cultured to 12 L fresh LB and incubated at 37 °C under agitation. When *A*_600nm_ reached 0.8, 1.0 mM IPTG was added, and the protein expression was induced at 20 °C overnight. Then, the protein was purified by Ni-affinity and gel filtration chromatography. The protein extinction coefficient was calculated by ProtParam, and the protein concentration was determined by the ultraviolet spectrophotometry method.

### 2.9. Biolayer Interferometry Assay 

In order to confirm whether the targeted protein aLDRG has the ability of chitinoligosaccharide binding, we conducted a biolayer interferometry (BLI) assay. Ligands, including *N*,*N*′-Diacetylchitobiose (CAS 35061-50-8), *N*,*N*′,*N*″-Triacetylchitotriose (CAS 38864-21-0), *N*,*N*′,*N*″,*N*′″-Tetraacetylchitotetraose (CAS 2706-65-2), *N*,*N*′,*N*″,*N*′″,*N*″″-Pentaacetylchitopentaose (CAS 36467-68-2) and chitooctaose (CAS 83143-57-1), were purchased from Shanghai Yuanye Bio-Technology Co., Ltd. (Shanghai, China) as the substrate ligands. aLDRG was biotinylated in assay buffer (phosphate-buffered saline with 0.01% Tween-20) for 30 min at room temperature (RT). The interaction between the ligand with aLDRG was determined by BLI assay using an Octet Red 96 instrument. Biotin-aLDRG was loaded to streptavidin biosensors by exposing biosensor tips in PCR tubes containing 15 μL biotin-aLDRG (50 μg/mL) for 20 min at RT. The binding data were collected at 30 °C. The experiments comprised three steps: (1) baseline for 120 s, (2) association for 120 s, (3) dissociation for 120 s. The double-reference-subtracted data globally fit 1:1 with Octet User software (version 9. 0) to reveal the binding constants. Visualize curves were established using GraphPad Prism 5.0.

## 3. Results

### 3.1. Armillaria sp. 541 RNA Sequences 

By performing comparative transcriptomic analysis, functional genes responsible for changes in morphological characteristics of the hyphae (AH) and invasive rhizomorphs (AR) of *Armillaria* sp. 541 were identified. After the removal of reads containing unknown nucleotides, low-quality reads and adapter-related sequences from 45.96 Gb of raw data, we obtained 45.46 Gb of clean reads, with average Q20 (percentage of bases with quality > 20 in clean reads), Q30 and GC percentages of 97.64%/97.59%, 93.40%/93.28% and 52.09%/52.04% for AH and AR, respectively (Supplementary Table S2). Moreover, based on a comparison of the clean reads of each sample with the reference genome sequence of *Armillaria gallica* (taxid47427) [18] using STAR software, we obtained 26.3 million unique reads. A list of the alignments and distribution of reads mapped to genomic regions are presented in Supplementary Table S3, and RNA sequencing data obtained in this study were uploaded to the NCBI database under accession number SUB8218370.

All the clean data were assembled using Cufflinks [27], and the Cuffcompare model was subsequently used for comparisons with *A. gallica* (taxid47427) genome sequences to detect novel genes. We accordingly identified a total of 1272 *Armillaria* sp. 541 unigenes that could not be annotated, which were sequentially numbered Novel00001, Novel00002, Novel00003, etc.

### 3.2. Differentially Expressed Genes 

**Figure 1:**
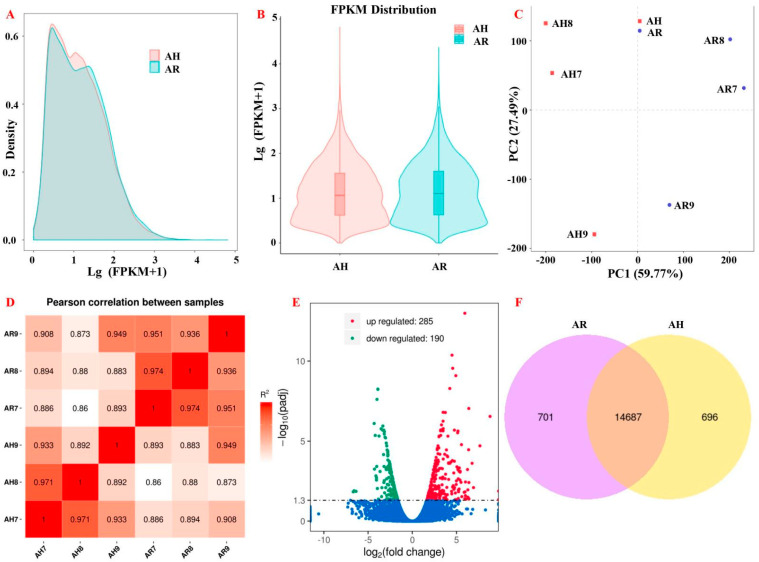
Data quality control and analysis of *Armillaria* sp. 541 transcriptome. The density distribution of genes with quantified value is no less than 1 (**A**). FPKM distributed between rhizomorphs (AR) and hypha (AH) (**B**). AR and AH were the significant groups determined by principal component analysis (PCA) (**C**) and the correlation between them also met the requirements (R^2^ > 0.8) via Pearson correlation tests (**D**). The differentially expressed genes (DEGs) were shown as a volcano map (**E**) and the specific genes in AR or AH were compared with the Venn diagram (**F**).

We succeeded in quantifying 27,293 unigenes in *Armillaria* sp. 541, the different expression levels of which are shown in Supplementary Table S4. The expression levels of the genes associated with AH and AR were compared by plotting all gene FPKM values as density distribution maps and violin plots (Figure 1A,B). PCA analysis results show the fact that the two treatments were clearly separate, and this also support the concept that rhizomorphs are distinct from hyphae physiologically (Figure 1C). Pearson correlation tests established the correlations among samples (Figure 1D).

Compared with AH, we identified a total of 475 unigenes that were differentially expressed in AR samples (fold change ≥ 2 or ≤0.5 and false discovery rate ≤ 0.05), among which 285 were upregulated and 190 were downregulated (Figure 1E). Furthermore, a total of 701 unigenes were uniquely expressed in AR, whereas 696 unigenes were only expressed in AH, and 14,687 expressed were in a similar manner in both AH and AR (Figure 1F).

### 3.3. Functional Annotation of DEGs

**Figure 2:**
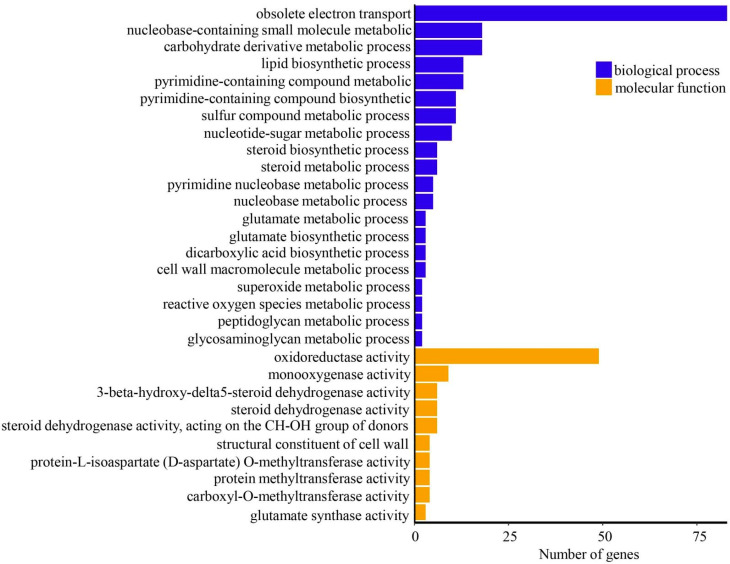
Proportions and Gene Ontology classifications of differentially expressed genes (top 20).

In order to elucidate functions, we annotated DEGs based on GO and KEGG enrichment analyses. Among biological processes, we identified the obsolete electron transport pathway as being significantly enriched, with 83 DEGs grouped in this subcategory (Figure 2). This is consistent with the analysis and enrichment results of the molecular function of GO. It revealed that 9 and 49 DEGs were, respectively, mapped to monooxygenase and oxidoreductase activities. These DEGs play roles in oxidoreductase activities, including acting on the sulfur group of donors, the CH-NH2 group of donors, the CH-OH group of donors, disulfide oxidoreductase activity, superoxide radicals as acceptors and NAD or NADP as acceptors (Figure 2). We also detected enrichment of the following genes in *Armillaria* sp. 541 rhizomorphs: 18 DEGs involved in the metabolic process nucleobase-containing small molecule; 18 in the metabolic process carbohydrate derivative; 13 in the biosynthetic process lipid; and 11 in the biosynthetic process pyrimidine-containing compound. These findings accordingly indicate that nucleotide, carbohydrate and lipid metabolic activity is activated concomitantly with the differentiation of AR from AH.

In this study, we focused on those genes in AR associated with reorganization function. We accordingly identified three unigenes differentially expressed between AR and AH that play roles in cell-wall macromolecule metabolic processes, namely *PBL01678*, *PBK99940* and *PBK94850* (Table 1). Gene Ontology classification results show that these three genes were annotated to peptidoglycan binding, cell-wall organization and biogenesis functions.

### 3.4. Validation of the Expression of the DEGs

By qRT-PCR, six DEGs expressed up in AR compared with AH harvested from the PDA medium. These results are consistent with the quantitative results by FPKM (Table 1), proving good accuracy for transcriptomic quantification. For targeted gene *PBL01678*, it was upregulated significantly with the ratio 3.47 (AR:AH) cultured on PDA medium. After the addition of diphenyleneiodonium chloride (DPI), an inhibitor of reduced nicotinamide adenine dinucleotide phosphate (NADPH) oxidase (NOX) [36], rhizomorph generation was inhibited, and the expression levels of *PBL01678* also downregulated remarkably with AR/AH 0.59. The expression of *PBL01678* was changed with the generation degree of rhizomorphs.

### 3.5. LysM Domain Proteins in Armillaria sp. 541 

Previous studies demonstrated that the LysM domain involved in binding peptidoglycan is a recognition function [24]. Therefore, we constructed a database of LysM domains of fungi and prepared a reference library, against which we screened the transcriptomic sequences of *Armillaria* sp. 541. We accordingly identified a total of 41 unigenes that putatively encode proteins containing a LysM domain. The domain(s) of these transcripts were further verified using Pfam. We identified 14 *Armillaria* sp. 541 unigenes encoding the LysM domain, the basic characteristics of which are listed in Table 2. All of these genes encoding the LysM domain, only four proteins in them contain two LysM domains, including PBL01701, PBL01678, PBK88887 and PBL01680. Other than PBL00098, PBK92036, PBK88887 and PBK92767, the rest contain one signal peptide (Supplementary Figure S1) and a βααβ structure in accordance with LysM effector (Table 2 and Supplementary Figure S2).

**Figure 3:**
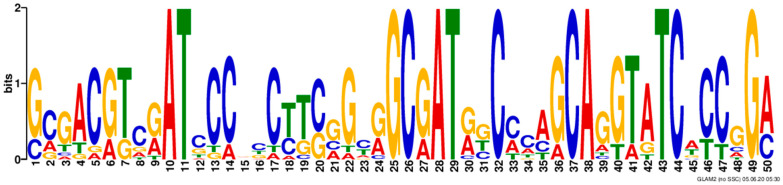
Consensus pattern analyses of *Armillaria* sp. 541 LysM motifs. Consensus pattern analysis of *Armillaria* sp. 541 LysM motifs generated using GLAM2 and GLAM2SCAN. These domains contain four conserved cysteines and conserved patterns for alanine (A), threonine (T) and glycine (G). The upper- and lower-case letters on the Y-axis mean frequency of occurrence, and the numbering 1–50 on the X-axis mean position information. Larger letters indicate a more conservative locus.

Subsequently, we performed gapped local alignments of *Armillaria* sp. 541 LysM motifs and generated consensus patterns using GLAM2 and GLAM2SCAN, respectively [33], which clearly revealed that the LysM domains in the *Armillaria* sp. 541 are characterized by four conserved cysteines (positions 26, 32, 37 and 44) (Figure 3), as well as a further three types of conserved pattern, with alanine (A) at positions 10, 28 and 38; threonine (T) at positions 11, 29 and 43; and glycine (G) at positions 25 and 49 (Figure 3). The presence of the four conserved cysteines tend to indicate that these motifs may be stabilized by disulfide bridges, whereas the other conserved residues may contribute to novel biological functions in *Armillaria* sp. 541.

**Figure 4:**
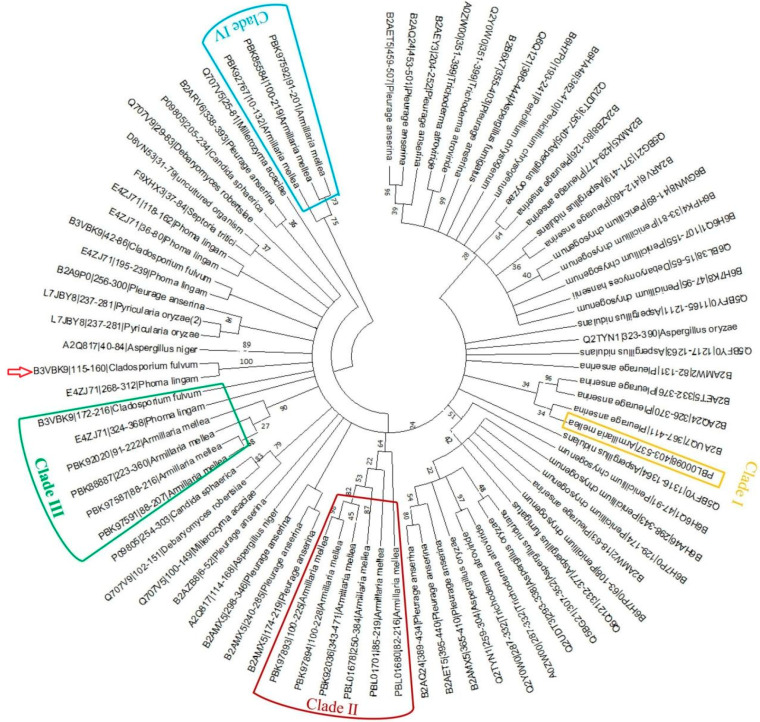
Phylogenetic analyses of *Armillaria* sp. 541 LysM motifs. Phylogenetic analysis of *Armillaria* sp. 541 LysM motif-containing proteins using MEGA 6.0. *Armillaria* sp. 541 LysM domain-containing proteins can be divided into four different clades, designated clades I to IV. The names of genes clustering in this clade comprise the protein ID, the LysM domain amino acid start and end sites, and species name. Red arrow indicates *Cladosporium fulvum*.

To further analyze the phylogenetic relationship of these LysM domain-containing proteins and the LysM domain-containing proteins in the reference fungal library, we used MEGA 11.0 [34], which revealed that LysMs encoded by these 14 unigenes clustered into four different clades, designated clades I to IV (Figure 4). In clade I, PBL00098 is closely related to B2AUQ7 (a putative glycoside hydrolase family 18) from *Pleurage anserina*, which has the molecular function of hydrolase activity, catalyzing the hydrolysis of *O*-glycosyl compounds. Uniprot annotation results revealed that the unigenes in clades II, III and IV cluster with proteins showing chitin-binding activity (Q707V5, the chitin-binding protein of *Millerozyma acacia*); hydrolase activity, hydrolyzing *O*-glycosyl compounds (Q2TYN1, an uncharacterized protein of *Aspergillus oryzae*); and chitinase activity (Q2Y0W0, chitinase 18-10 of *Trichoderma atroviride*).

On the basis of these analyses, we thus speculate that PBL01678 rather than PBK99940 and PBK94850 might be more likely to perform cell-wall binding via the LysM domain.

### 3.6. In Situ Hybridization of PBL01678 

**Figure 5:**
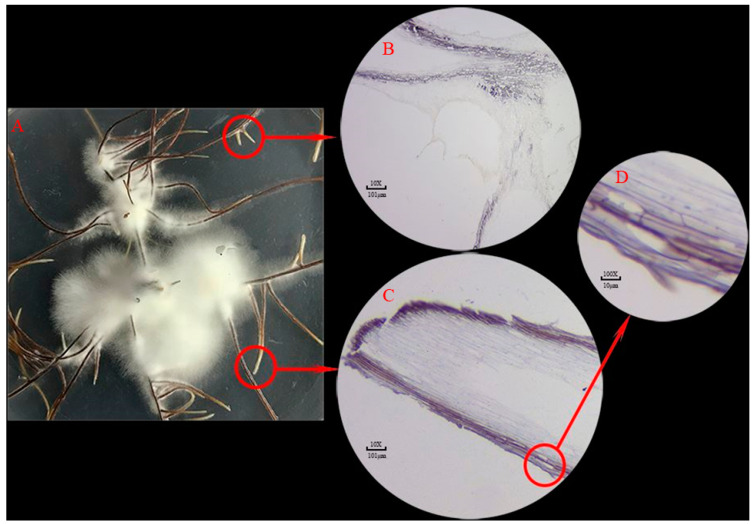
In situ hybridization analysis of the *aLDRG*. (**A**) presents the morphological characteristics of *Armillaria* sp. 541 cultured on PDA. *PBL01678* are labeled on mature sections (**B**) and white growth tips of rhizomorphs (**C**,**D**), in which (**D**) is a partial enlargement of (**C**).

There was no annotation information for *PBL01678* following the GO and KEGG analysis. To further investigate the potential roles of *PBL01678* during rhizomorph generation, we performed in situ hybridization to examine expression patterns, as well as real-time quantitative PCR (qRT-PCR). The molecular weight of the probe was 9082.7, and the difference between actual molecular weight and theoretical molecular weight was less than 0.3% (Supplementary Table S5). Labeling with a digoxin probe revealed that *PBL01678* was located in one to three cell layers beneath the epidermis of the rhizomorph. Our observations tended to indicate that rather than being characterized by site-specific localization, *PBL01678* is widely distributed throughout the cortical layers of rhizomorphs, including both the mature sections (Figure 5B) and white growth tips (Figure 5C,D).

### 3.7. LysM Domain of Protein aLDRG Binds to Chitinoligosaccharide

**Figure 6:**
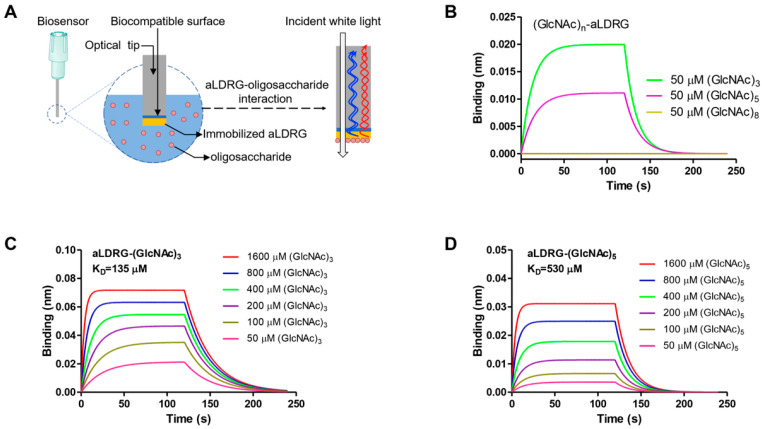
Biolayer interferometry (BLI) assay between aLDRG and chitinoligosaccharides. Schematic diagram for BLI assay (**A**). (**B**) BLI experiments of aLDRG binding to oligosaccharide. A concentration of 50 μM was used for each analyte. (**C**,**D**) Binding of (GlcNAc)3 or (GlcNAc)5 to immobilized biotinylated aLDRG. A concentration range of analyte (50 to 1600 μM) was used for each experiment.

To verify whether protein PBL01678 has the ability to bind to the cell-wall substrate, recombinant PBL01678 expressed in *E*. *coli* BL21 (DE3) was purified and its affinity constant for chitinoligosaccharide was determined by BLI assay (Figure 6A). Protein PBL01678 is a type of secreted protein. The BLI assay demonstrated that the PBL01678 protein can bind to *N*,*N*′,*N*″-Triacetylchitotriose (CO3) and *N*,*N*′,*N*″,*N*′,*N*″″-Pentaacetylchitopentaose (CO5) specifically, rather than *N*,*N*′-Diacetylchitobiose (CO2), *N*,*N*′,*N*″,*N*′″-Tetraacetylchitotetraose (CO4) or chitooctaose (CO8) (Figure 6B and Supplementary Table S6). The PBL01678 bound CO3 and CO5 with an increasing affinity from 50 to 1600 μM with K_D_ 135 μM and 530 μM, respectively (Figure 6C,D, Supplementary Tables S7 and S8). The results demonstrate that PBL01678 can selectively recognize specific oligosaccharide of chitin in the fungal cell wall. Therefore, the PBL01678 protein was named the *Armillaria LysM domain-recognition gene* (*aLDRG*) temporarily.

## 4. Discussion

In general, *Armillaria* spp. are well-known pathogens causing large economic losses throughout the world. However, some *Armillaria* species such as *Armillaria mellea* can establish a symbiotic relationship with *G. elata* or *P. umbellatus*, both of which are widely used as TCM in Asia [3,4]. In the natural condition, *Armillaria* sp. 541 does not eliminate *P. umbellatus* sclerotia, but establishes a symbiotic relationship with this medicinal fungus [24]. During this process, rhizomorphs play an important role. Since little is known about the complexity of symbiotic *Armillaria* rhizomorphs, RNA sequencing and analysis help us better understand the differentiation area of rhizomorphs and obtain much more functional genes and bio-information.

There are few examples of research and reports on rhizomorph generation, for example, the differentiation and branching of rhizomorphs were reported to be related to calcium ion flux. Transcriptomic tests and DEG analysis confirmed the complexity of rhizomorph formation from hyphae of *Armillaria* sp. Large numbers of DEGs were identified as being involved in biological or metabolic pathways attributed to rhizomorphs generation, such as *PBK98490* in oxidoreductase activity, *PBK99671* in the carbohydrate metabolic process and *PBL04375* in the oxalate metabolic process (Table 1). Gene Ontology enrichment results indicated that oxidoreductase activity was closely associated with rhizomorph formation, and this is consistent with differential proteomic results [40]. It has previously been reported that fungal NADPH oxidase (Nox) complexes are involved in the fungal differentiation necessary for phytopathogenicity [41]. Our previous study demonstrated that DPI, a specific inhibitor of the ROS-generating enzyme Nox, can inhibit the generation of rhizomorphs of *Armillaria* sp. 541 significantly, and the number of rhizomorphs (22 ± 8) in the added DPI group was only half of that in the control group (46 ± 12) [42]. In addition, further research pointed out that thioredoxin-like protein promotes H_2_O_2_ signaling and oxidative stress resistance [43]. After adding DPI, *PBK98490* encoding thioredoxin-like protein functioned in oxidoreductase activity–antioxidant activity downregulated remarkably with an AR_(DPI)_:AH_(DPI)_ ratio 1.27, while there was an AR:AH ratio 9.88 in the control group (Table 1). These observations thus indicate that ROS production is the key element associated with rhizomorph generation in *Armillaria* sp. 541. It was reported that thioredoxin took part in melanin production in bacteria through the regulation of laccase or tyrosinase activities [44]. After rhizomorph was generated, the melanin layer gradually formed. Thus, both rhizomorph generation and structure might be closely regulated by oxidative stress. Other biological processes, like copper ion binding, also contributed to rhizomorphs’ generation. Three genes expressed differentially between AR and AH, including *PBK89196*, *PBK82625* and *PBK80004* encoding laccase, phenol oxidase and Cu-oxidase domain-containing protein. Dedeyan et al. [45] found that white rot fungus *Marasmius quercophilus* could also generate rhizomorphs by colonizing the surface of leaves, resulting in the complete bleaching of the leaf. They purified laccase from this fungus and proved it belongs to the “blue copper oxidase” family and degraded humus. Gene *PBK89196* was upwardly expressed with a ratio 3.37 (AR/AH, quantified by FPKM), indicating that the genes involved in copper ion binding might contribute to rhizomorph for digesting humus.

Genes functioning in cell-wall binding were other essential types of genes during rhizomorphs’ generation from hyphae. The establishment of a symbiotic relationship between fungi and their hosts is a mutually beneficial process, providing beneficial effects to their hosts while gaining advantages [46]. Some types of *Armillaria* sp. can also invade *P. umbellatus* sclerotia and form symbiont [13], in which rhizomorphs are the functional tissue. It was reported that the rhizomorphs were formed to withstand adverse environments, search for nutrients and penetrate hosts [3,10,12]. However, what might the functional genes be? It was found that the LysM domain was reported to play a crucial role in fungi interaction with the host via binding to the chitinoligosaccharide of the cell wall [23]. A screen of the *Armillaria* sp. 541 transcriptome revealed 14 unigenes encoding LysM domain-containing proteins (Table 2). These LysM share evolutionally conserved sites, including alanine (A), threonine (T) and glycine (G), suggesting that their main biological functions might be similar to those reported in other species, such as *Cladosporium fulvum* (red arrow in Figure 4). *C. fulvum* is a serious pathogen that can cause tomato leaf mold in which Ecp6 is the crucial effector including three LysMs. LysM1 and LysM3 can form a dimer to form a chitin-binding site with high affinity, which is higher than the host receptor, resulting in the inhibition of the host defense response and infection. The silencing of *Ecp6* led to a significant reduction in tomato disease infected by the mutants compared to wild strains [47,48]. In this study, *aLDRG* was one of these genes significantly upregulated in rhizomorphs confirmed by qRT-PCR compared with hyphae, suggesting that the formation of rhizomorphs might be responsible for this protein. Functionally, the LysM domain of the host also plays a role in the recognition fungi or fungal LysM effector, which enhances the infection ability of fungi by binding of (GlcNAc)n (a subunit of the cell wall) [49,50]. In order to confirm whether aLDRG (containing two LysM domains in Table 2) has the ability to bind to GlcNAc oligomers, the affinity test confirmed that the recombinant aLDRG of *Armillaria* sp. 541 can bind to *N*,*N*′,*N*″-triacetylchitotriose (CO3) and *N*,*N*′,*N*″,*N*′″,*N*″″-pentaacetylchitopentaose (CO5), the units of cell-wall components in fungi accordingly (Figure 6). These results confirmed that *Armillaria* sp. can release much more LysM proteins, such as aLDRG, after the rhizomorphs differentiated from hyphae. In general, fungal LysM competes with host plant LysM for chitin oligomers’ binding. For example, *Magnaporthe oryzae* can secret LysM protein Slp1 (secreted LysM protein1), which can competitively bind chitin to rice chitin elicitor-binding protein (OsCEBiP) (including two LysM domains), thus inhibiting chitin-induced plant immune response [51]. Over-expressed LysM might bind to more chitin oligomers, helping *Armillaria* rhizomorphs evade host recognition, thereby weakening the chitin-induced signaling pathway, the host’s immune system against fungus invasion. Our study has shown that by analyzing transcriptome results, a series of genes was altered in expression during rhizomorph differentiation of *Armillaria* sp. 541, with some of the LysM-containing proteins shown to be able to interact with chitin and possibly be involved in interactions with the host. The detailed mechanism of this type of protein in the interaction between *Armillaria* sp. 541 and its host needs to be further studied. It will also be useful to provide some reference for the pathogenic mechanisms and control methods of some *Armillaria* spp., such as forest and crop pathogens.

## Figures and Tables

**Table 1:** The expressed ratios of targeted genes in rhizomorphs and hyphae of *Armillaria* sp. 541.

Gene.	Encoding Protein	Gene Description	Ratio by FPKM	Ratio by qRT-PCR	
			AR/AH of CK Group	AR/AH of DPI Group	AR/AH of CK Group
*PBK98490*	thioredoxin-like protein	acts as a virulence effecter; oxidoreductase activity–antioxidant activity	9.88	1.27	3.24
*PBL01678*	LysM domain recognition protein	contains lysine motif, which is a small domain involved in binding peptidoglycan, cell-wall organization or biogenesis	8.21	0.59	3.47
*PBL01750*	alpha/beta-hydrolase	abhydrolase_4 activity	13.4	2.00	0.54
*PBK99671*	D-xylulose 5-phosphate/D-fructose 6-phosphate phosphoketolase	lyase activity, polysaccharide lyases	9.67	1.23	2.24
*PBK94850*	glycoside hydrolase family 25 protein	lysozyme activity; cell-wall catabolic process-peptidoglycan catabolic process	8.01	1.06	4.71
*PBL03369*	MFS general substrate transporter	MFS general substrate transporter; carbohydrate transport and metabolism	6.59	0.96	2.34
*PBL04375*	oxalate decarboxylase	nutrient reservoir activity	5.96	0.55	5.36

**Table 2:** Characteristics of proteins containing LysM domain of *Armillaria* sp. 541.

Protein.	Numbers of Amino Acids	Molecular Weight (kDa)	pI	Hydropathicity	Signal Peptide (Sec/SPI)	LysM Domains
PBK97591	228	19.1	5.30	0.753	0.9982	1
PBK97893	234	19.7	5.29	0.812	0.9984	1
PBK85584	234	20.0	5.27	0.926	0.9983	1
PBK92020	234	20.1	5.27	0.850	0.9978	1
PBK97587	252	21.0	5.29	0.731	0.9986	1
PBK97894	279	23.5	5.25	0.882	0.9984	1
PBK92767	387	32.1	5.20	0.966	0.0017	1
PBL01678	408	34.5	5.20	0.755	0.9816	2
PBL01701	408	34.5	5.19	0.881	0.9894	2
PBL01680	408	35.0	5.22	0.706	0.9967	2
PBK88887	435	36.8	5.16	0.980	0.0452	2
PBK92036	483	39.7	5.18	0.908	0.0010	1
PBL00098	555	45.9	5.12	0.927	0.0014	1
PBK97592	675	56.7	5.09	0.942	0.9987	1

## Data Availability

The unigene database of *Armillaria* sp. 541 has been deposited in NCBI (https://www.ncbi.nlm.nih.gov/, accessed on 28 April 2023) under the accession number SUB8218370.

## Supplementary Materials

The following supporting information can be downloaded at: https://www.mdpi.com/article/10.3390/microorganisms11081914/s1, Table S1: The qRT-PCR primers of genes validated in paper; Table S2: Sequencing data statistics; Table S3: The alignment summary of *Armillaria* sp. 541 reads compared with reference *A. gallica* (taxid47427); Table S4: The statistic results of the numbers of genes in different expression levels for *Armillaria* sp. 541 AH and AR; Table S5: Probe information for targeted gene; Table S6: Excel Report of aLDRG binds to GlcNAc2 3 4 5 8; Table S7: Excel Report of aLDRG binds to GlcNAc3; Table S8: Excel Report of aLDRG binds to GlcNAc5.
