# Supplementary material for: Hallmarks of Comparative Transcriptome between Rhizomorphs and Hyphae of Armillaria sp. 541 Participating in Fungal Symbiosis with Emphasis on LysM Domains
Source: Microorganisms. 2023 Jul 27;11(8):1914. doi: 10.3390/microorganisms11081914 (PMC10458900; doi:10.3390/microorganisms11081914)
Supplement: Supplementary file 1 [file microorganisms-11-01914-s001.zip › Supplementary Table S1-S4.pdf]

Table S1 The qRT-PCR primers of genes validated in paper

Gene ID	Primer sequence 5'-3'
PBK98490	AGGCAGAGACGACGCTAGGCAA
	GGTGTTCCTCAAGACCGTTCGC
PBL01678	CTGTGCTCGCAATTACACCG
	CTGGGTATTCACCGAAGCGA
PBL01750	GAGACGGTTGTGCTCGCCATCA
	TCGTTCAGCGGCCAACTGTCCA
PBK99671	GCCACATCAATGCCGCAACTCC
	GACAACGCAGGCGACGACAAGA
PBK94850	GAATCAGGTTGTCGCGAACG
	AGGGTGGGCGAAATGGTATG
PBL03369	GGAGAACTTGGGCAAGCGGTGT
	ACGGAGGAAGAGGCGGTCAGAA
PBL04375	GGATGACGAAGGCGGCTCTGAA
	CCTCGGCAACGGCGATTGTAGT
EF-1γ	TGCTCGGCTCGACTCCAGAAGA
	TGGCACCGCATTGGATGAAGGC

Table S2 Sequencing data statistics

Sample	Raw Reads	Raw Bases	Clean Reads	Clean Bases	Error Rate (%)	Q20 (%)	Q30 (%)	GC Content (%)
**AH7_1**	26899251	4.03G	26574075	3.99G	0.03	97.91	94.13	52.16
**AH7_2**	26899251	4.03G	26574075	3.99G	0.03	97.28	92.50	52.17
**AH8_1**	28873379	4.33G	28608034	4.29G	0.03	97.91	94.13	52.06
**AH8_2**	28873379	4.33G	28608034	4.29G	0.03	97.42	92.80	52.08
**AH9_1**	22732540	3.4G	22518707	3.38G	0.03	97.91	94.14	52.03
**AH9_2**	22732540	3.4G	22518707	3.38G	0.03	97.38	92.72	52.05
**AR7_1**	26561842	3.98G	26104985	3.92G	0.03	97.97	94.26	52.08
**AR7_2**	26561842	3.98G	26104985	3.92G	0.03	97.34	92.57	52.07
**AR8_1**	25723026	3.85G	25447458	3.82G	0.02	97.97	94.27	52.05
**AR8_2**	25723026	3.85G	25447458	3.82G	0.03	96.99	91.85	52.05
**AR9_1**	22632838	3.39G	22218084	3.33G	0.03	97.92	94.15	51.99
**AR9_2**	22632838	3.39G	22218084	3.33G	0.03	97.32	92.56	52.00

Table S3 The alignment summary of *Armillaria* sp. 541 reads compared with reference *A. gallica* (taxid47427)

**Sample name**	**AH7**	**AH8**	**AH9**	**AR7**	**AR8**	**AR9**
**Total reads**	53148150	57216068	45037414	52209970	50894916	44436168
**Total mapped**	47469446 (89.32%)	51201972 (89.49%)	39871508 (88.53%)	46889602 (89.81%)	45958090 (90.3%)	39617658 (89.16%)
**Multiple mapped**	1335538 (2.51%)	1413744 (2.47%)	1101164 (2.44%)	1205638 (2.31%)	1165846 (2.29%)	1056336 (2.38%)
**Uniquely mapped**	46133908 (86.8%)	49788228 (87.02%)	38770344 (86.08%)	45683964 (87.5%)	44792244 (88.01%)	38561322 (86.78%)
**Read-1**	23066954 (43.4%)	24894114 (43.51%)	19385172 (43.04%)	22841982 (43.75%)	22396122 (44%)	19280661 (43.39%)
**Read-2**	23066954 (43.4%)	24894114 (43.51%)	19385172 (43.04%)	22841982 (43.75%)	22396122 (44%)	19280661 (43.39%)
**Reads map to '+'**	23066954 (43.4%)	24894114 (43.51%)	19385172 (43.04%)	22841982 (43.75%)	22396122 (44%)	19280661 (43.39%)
**Reads map to '-'**	23066954 (43.4%)	24894114 (43.51%)	19385172 (43.04%)	22841982 (43.75%)	22396122 (44%)	19280661 (43.39%)
**Non-splice reads**	25739051 (48.43%)	28137464 (49.18%)	21559886 (47.87%)	23974840 (45.92%)	23676223 (46.52%)	20386412 (45.88%)
**Splice reads**	20394857 (38.37%)	21650764 (37.84%)	17210458 (38.21%)	21709124 (41.58%)	21116021 (41.49%)	18174910 (40.9%)
**exonic**	94.90%	94.78%	94.38%	95.44%	95.63%	95.14%
**intronic**	0.04%	0.04%	0.05%	0.05%	0.05%	0.05%
**intergenic**	5.06%	5.18%	5.57%	4.51%	4.32%	4.81%

Table S4 The statistic results of the numbers of genes in different expression levels for *Armillaria* sp. 541 AH and AR

FPKM Interval	AH7	AH8	AH9	AR7	AR8	AR9
**0~1**	12352 (45.26%)	12478 (45.72%)	11784 (43.18%)	12133 (44.45%)	12548 (45.98%)	11575 (42.41%)
**1~3**	3246 (11.89%)	3309 (12.12%)	2837 (10.39%)	3089 (11.32%)	3150 (11.54%)	2960 (10.85%)
**3~15**	5232 (19.17%)	5301 (19.42%)	5363 (19.65%)	5062 (18.55%)	4845 (17.75%)	5220 (19.13%)
**15~60**	3940 (14.44%)	3824 (14.01%)	4649 (17.03%)	4303 (15.77%)	4027 (14.75%)	4794 (17.56%)
**>60**	2523 (9.24%)	2381 (8.72%)	2660 (9.75%)	2706 (9.91%)	2723 (9.98%)	2744 (10.05%)
