# Supplementary figures and images for: Hallmarks of Comparative Transcriptome between Rhizomorphs and Hyphae of Armillaria sp. 541 Participating in Fungal Symbiosis with Emphasis on LysM Domains
Source: Microorganisms. 2023 Jul 27;11(8):1914. doi: 10.3390/microorganisms11081914 (PMC10458900; doi:10.3390/microorganisms11081914)

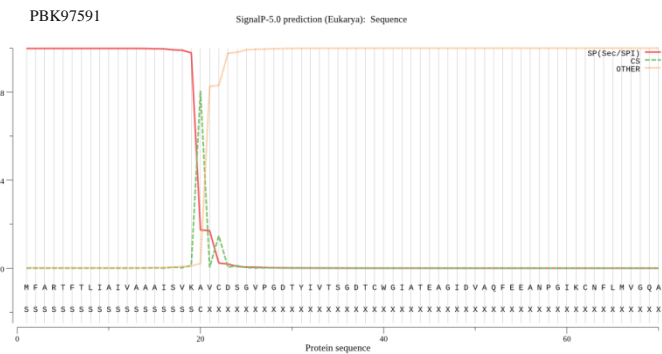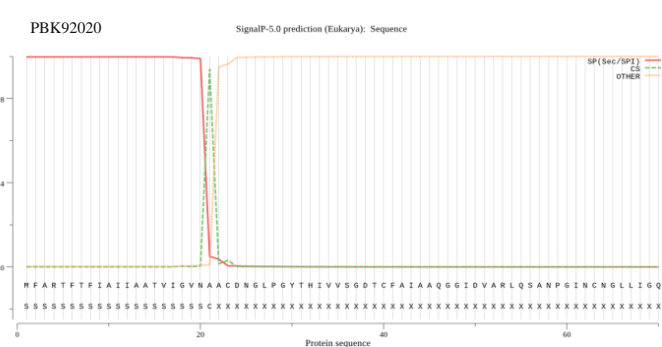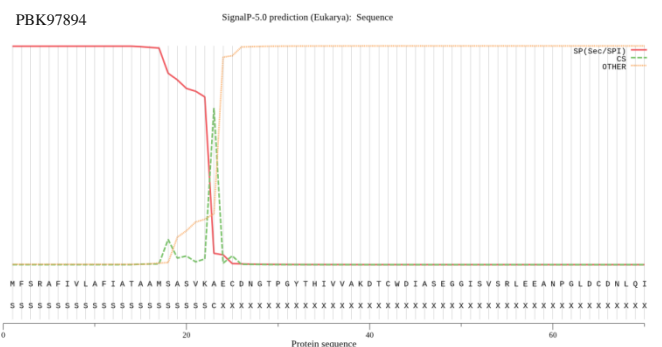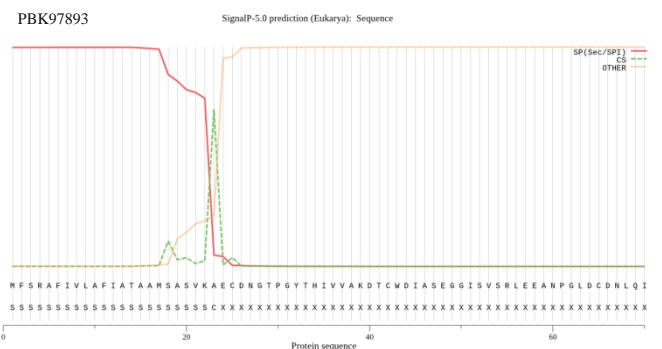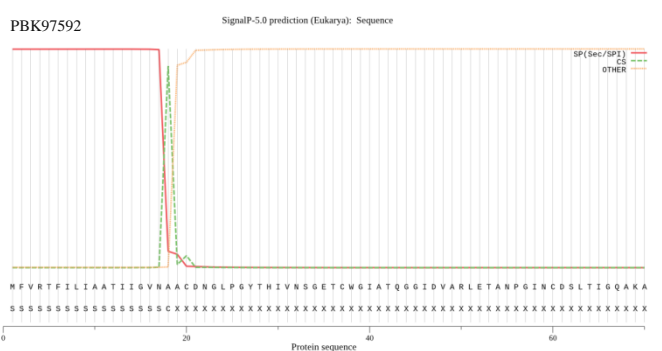

Supplement: Supplementary file 1 [file microorganisms-11-01914-s001.zip › Supplementary Figure S1.pdf]

|                                                                                     |                                                                                     |                                                                                     |                                                                                      |
|-------------------------------------------------------------------------------------|-------------------------------------------------------------------------------------|-------------------------------------------------------------------------------------|--------------------------------------------------------------------------------------|
| PBK85584                                                                            | PBK97591                                                                            | PBK97587                                                                            | PBL00098                                                                             |
| 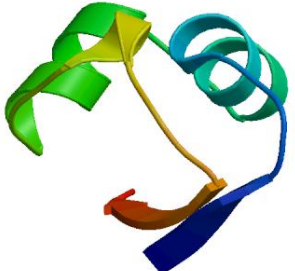   | 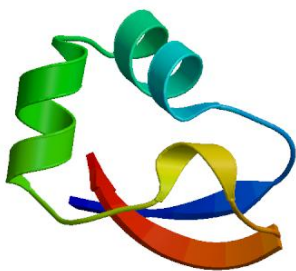   | 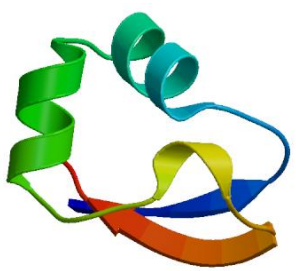  | 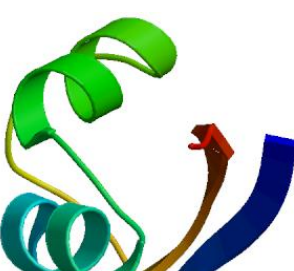  |
| PBK92020                                                                            | PBK92036                                                                            | PBL01701                                                                            | PBK97894                                                                             |
| 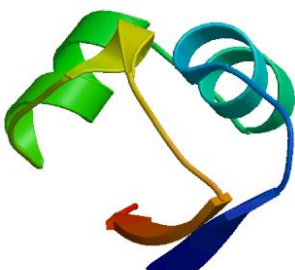   | 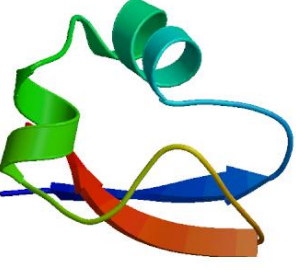   | 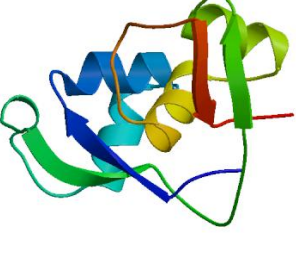  | 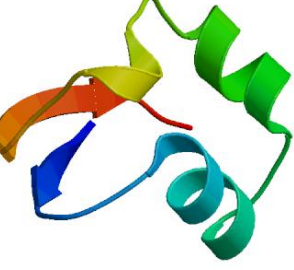  |
| PBL01678 (aLDRG)                                                                    | PBK97893                                                                            | PBK88887                                                                            | PBL01680                                                                             |
| 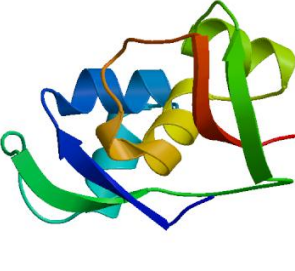  | 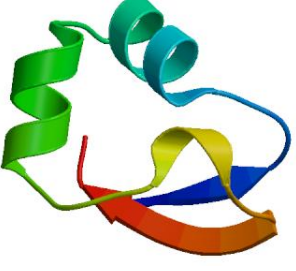  | 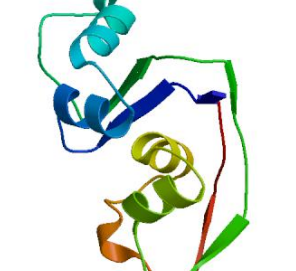 | 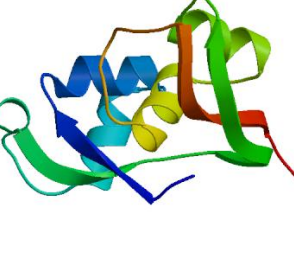 |
| PBK92767                                                                            | PBK97592                                                                            |                                                                                     |                                                                                      |
| 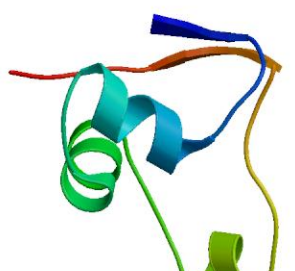 | 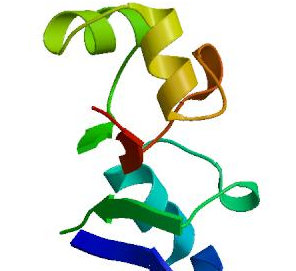 |                                                                                     |                                                                                      |

Supplement: Supplementary file 1 [file microorganisms-11-01914-s001.zip › Supplementary Figure S2.pdf]
